# Liver Enzymes in a Cohort of Community-Dwelling Older Persons: Focus on Sex Contribution

**DOI:** 10.3390/nu14234973

**Published:** 2022-11-23

**Authors:** Evelyn Ferri, Paolo D. Rossi, Martina Scichilone, Tiziano A. Lucchi, Beatrice Arosio

**Affiliations:** 1 Fondazione IRCCS Ca’ Granda Ospedale Maggiore Policlinico, Via Pace 9, 20122 Milan, Italy; 2 Department of Clinical Sciences and Community Health, University of Milan, Via Pace 9, 20122 Milan, Italy

**Keywords:** liver, sex, aging, dementia, alanine aminotransferase, aspartate aminotransferase

## Abstract

Dysfunctions in liver metabolic activities may increase the risk of cognitive impairment and dementia. In a cohort of community-dwelling older persons investigated for a suspected cognitive decline, we studied the association between liver status and dementia, considering sex and frailty contribution. Serum alanine aminotransferase (ALT) and aspartate aminotransferase (AST) concentrations, and the AST/ALT ratio were used to assess liver function in 419 older adults (248 persons with dementia and 171 age- and sex-matched subjects without cognitive decline). Although the serum concentrations of the liver enzymes were in the physiologic range, patients with dementia showed lower ALT concentrations (*p* = 0.005) and higher AST/ALT ratios (*p* = 0.003) compared to controls. The same differences were found when comparing men with and without dementia (ALT, *p* = 0.009; AST/ALT ratio, *p* = 0.003) but disappeared in women. Curiously, comparing women and men with the same diagnosis, the ALT concentrations were lower (*p* = 0.008), and the AST/ALT ratio was higher (*p* = 0.001) in control women than men, whereas no significant difference was found between persons with dementia. In conclusion, in our cohort of older people living in the community, the association between serum aminotransferases and dementia was remarked. Moreover, our results support attention to sex difference in liver function, suggesting a role in the pathogenesis of dementia.

## 1. Introduction

In 2022, the World Health Organization counted more than 55 million people living with dementia worldwide, and nearly 10 million new cases occur every year. Due to the increase in older people in almost every country, this number is expected to rise to 78 million in 2030 and 139 million in 2050 [1]. Dementia is the seventh leading cause of death among all diseases and one of the major causes of disability and dependency among people aged 65 and older [1]. Mounting evidence shows that the prevalence of dementia is higher in women than men, with 1.7 times more women with dementia than men in 2019 [2].

The most common forms of dementia affecting older people are Alzheimer’s disease (AD) and vascular dementia. However, the boundaries between the different forms of dementia are often blurred in older persons, and most people share the presence of both neurodegenerative and vascular features in the brain, resulting in a mixed dementia (MD) [3,4].

Numerous studies have revealed that metabolic dysfunctions (e.g., alterations in energy metabolism, metabolic signaling, and insulin resistance) may increase the risk of dementia [5,6,7]. In this context, the liver is one of the major metabolic hubs, and metabolic activities in the liver determine the state of the metabolic readout of peripheral circulation. Dysfunctions in the hepatic metabolic activities are reported to contribute to cognitive impairment and dementia [8,9,10,11,12,13].

To assess liver function and measure liver injury, serum alanine aminotransferase (ALT) and aspartate aminotransferase (AST) concentrations are widely used in clinical practice [14,15]. ALT catalyzes a reaction between alanine and α-ketoglutarate to produce pyruvate and glutamate, whereas AST catalyzes a reaction between aspartate and α-ketoglutarate to produce oxaloacetate and glutamate [16]. ALT is mainly found in the cytosol of hepatocytes, thus it is considered the most liver-specific enzyme. In contrast, AST is synthesized not only in the liver but also in skeletal muscle, the heart, the brain, and other tissues of the body. The AST to ALT ratio (De Ritis ratio) is an indicator of liver function and prognosticates the severity of hepatic disease [17,18].

There is some evidence that low serum concentrations of liver enzymes, in particular ALT, are a biomarker for sarcopenia, malnutrition, frailty, and disability [19,20,21,22,23]. Moreover, many studies have associated raised levels of ALT with insulin resistance, a metabolic syndrome, and type 2 diabetes [24,25,26], and sex differences in these liver-associated metabolic adaptations have clearly been illustrated [27,28,29]. Interestingly, a strong age dependence of ALT concentrations was shown in men, whereas this difference was attenuated in women [30].

The liver is known to have a pivotal role in the metabolism of toxic compounds such as drugs or alcohol, but when exposure to large amount of these compounds becomes chronic, hepatic pathologies can be induced, and the release of several harmful substances (e.g., ammonium and pro-inflammatory cytokines) may be promoted [31]. For instance, the release of these toxic compounds promotes the development of hepatic encephalopathy, a common and debilitating neuropsychiatric complication of chronic liver disease [32,33,34]. In hepatic encephalopathy, the liver failure is accompanied by neuroinflammation, induced by transport through the systemic circulation and the accumulation in the brain of ammonia and pro-inflammatory cytokines [35,36,37,38], which trigger the reactivity of microglia, promote the recruitment of monocytes, and alter the permeability of the blood–brain barrier [39].

A recent study conducted in a mouse model of alcohol-induced liver disease confirmed that chronic alcohol intake increases peripheral as well as brain alcohol levels, leading to an increase in ALT production in the serum as well as neuroinflammation that seems to cause memory impairment and reduce sensorimotor coordination [12].

Interestingly, growing evidence suggests a key role of liver function and, in particular, liver hypometabolism (indexed by a reduction in ALT and AST synthesis) also in the pathophysiology of AD [8]. Indeed, lower ALT levels in serum and higher AST/ALT ratio values were found in patients with AD compared to subjects without cognitive decline [8,40]. Reduced levels of ALT have been associated with reduced brain glucose metabolism, cerebral atrophy, brain amyloid-β deposition, and alterations in neurodegenerative biomarkers concentrations in the cerebrospinal fluid of persons with AD [8].

In light of these premises, we characterized the liver status, investigating the AST and ALT concentrations and AST/ALT ratio values in a cohort of older persons living in the community investigated for a suspected cognitive decline. In particular, as sex differences in liver-associated metabolic functions are known, our aim was to explore the association between hepatic enzymes and dementia, evaluating the contribution of sex and frailty.

## 2. Materials and Methods

### 2.1. Study Design

The cohort consisted of 419 community-dwelling older adults (65–93 years) who consecutively underwent a first geriatric visit to the Geriatric Unit of the Fondazione IRCCS Ca’ Granda Ospedale Maggiore Policlinico of Milan (Italy) from March 2009 to February 2018.

All participants were admitted to investigate a suspected cognitive decline. A multidimensional geriatric assessment provided information on their medical history and cognitive, functional, and physical status. Relatives often participated in the geriatric visit by providing additional information about the subject’s medical history. The collected data were recorded in “Registro di Raccolta Dati della Unità di Geriatria” (REGE 2.0).

A modified version of the mini mental state examination (MMSE) was used to assess the cognitive status of all subjects involved in the study [41]. A neuropsychological assessment battery (i.e., trail making test, verbal fluency test, digit span forward and backward tests, verbal learning tests, token test, Rey’s figure copy and delayed recall, Raven’s colored progressive matrices) was administered in case of MMSE < 24. The geriatric depression scale (GDS) was used to evaluate the presence of depression.

For each participant, the body mass index (BMI) was calculated. Persons with BMI < 18.5 kg/m^2^ were considered underweight, 18.5 kg/m^2^ ≤ BMI < 25 kg/m^2^ normal, 25 kg/m^2^ ≤ BMI < 30 kg/m^2^ pre-obese, and BMI ≥ 30 kg/m^2^ obese [42].

The cohort consisted of 248 persons with dementia comprising 65 AD and 183 MD patients and 171 age- and sex-matched subjects without cognitive decline (controls). The diagnosis of AD was made according to the criteria by Dubois et al. [43], whereas individuals showing an overlap between neurodegenerative features (hippocampal atrophy and markers of neurodegeneration) and vascular brain injury (cerebral microangiopathy and infarcts) were diagnosed as MD [44,45]. Controls were subjects with a MMSE score ≥ 24 and absence of neurological or psychiatric disorders. These subjects were examined once a year for up to five years to assess their cognitive status and ensure that they kept their cognitive abilities intact.

Subjects who reported a past or current history of abuse of and/or dependence on alcohol were not considered for this study.

All the participants gave informed consent personally or through their legal guardian (if the person was mentally incapable of making an autonomous decision) for their clinical and biological data to be used for properly anonymized research purposes.

The study was conducted in accordance with the Declaration of Helsinki, and the research protocol received approval from the Ethics Committee of the Fondazione IRCCS Ca’ Granda Ospedale Maggiore Policlinico of Milan (REGE 2.0, protocol number 1696, 4 June 2021).

### 2.2. Frailty Index

Frailty was measured through the frailty index (FI) [46]. The FI results from the count of various health deficits, including signs, diseases, disabilities, and biochemical parameters, as previously described [47].

Briefly, each deficit was dichotomized and scored as ‘0’ (absence of the deficit) or ‘1’ (presence of the deficit). The FI of each subject included in the study was calculated as the total number of health deficits divided by the total number of variables considered for its computation (*n* = 47) (Table S1).

The FI is measured on a continuous scale, which can be divided into severity levels to define the different health status. Rockwood et al. found that frailty was defined as FI > 0.25 [48,49].

### 2.3. Determination of Serum Liver Enzyme Concentrations

Serum AST and ALT concentrations were measured using the method recommended by the International Federation of Clinical Chemistry (IFCC) with pyridoxal phosphate activation at 37 °C with the Cobas c 702 Analyzer (Roche, Basel, Switzerland).

The normal range of values for AST in serum was 10–33 U/L in women and 10–35 U/L in men. In comparison, the normal range of values for ALT in serum was 6–41 U/L in women and 9–59 U/L in men. The AST to ALT ratio (AST/ALT ratio) is the ratio between the concentrations of AST and ALT enzymes.

### 2.4. Statistical Analysis

The statistical analyses were conducted using IBM SPSS Statistic software (version 27, IBM Inc., Chicago, IL, USA).

The distribution of clinical and biochemical parameters was assessed using Kolmogorov–Smirnov test to investigate the adherence to the Gaussian graph. Normally distributed variables (age, education, MMSE, GDS, and BMI) were reported as mean and standard deviation (SD), whereas non-normally distributed variables (FI, AST and ALT concentrations, and AST/ALT ratio) were expressed as median and interquartile range (IQR: 25–75th percentile).

The normally distributed variables were analyzed using the Student’s *t*-test and the analysis of variance (ANOVA), followed by Bonferroni post-hoc test, whereas the non- normally distributed variables were examined using the Mann–Whitney U test and the Kruskal–Wallis test.

FI, AST, and ALT concentrations and AST/ALT ratio values were log-transformed for regression analyses. Multivariate linear regression analyses were assessed to investigate the association between liver enzyme parameters and MMSE scores, after adjustment for age, FI, and BMI as confounding factors. The regression results were expressed as unstandardized beta (B) coefficient, followed by the standard error for the unstandardized beta (SE(B)).

Values of *p* < 0.05 were considered statistically significant.

## 3. Results

In the overall cohort, the percentage of women was 70.2%. The FI values ranged from 0.04 to 0.69. Regarding the liver parameters, the AST concentrations ranged from 7.0 to 45.0 U/L, ALT concentrations from 5.0 to 43.0 U/L, and AST/ALT ratio values from 0.58 to 4.75.

Categorizing the subjects by sex, no difference was found in the age, MMSE score, and BMI between women and men (Table 1). As expected, women showed a lower educational level and a higher GDS score than men, but they were less frail compared to men (Table 1).

Regarding liver enzymes, the AST levels were comparable between the groups. Contrarily, women showed lower ALT levels and higher AST/ALT ratio values than men (Table 1).

The multivariate linear regression analyses adjusted for age, FI, and BMI showed associations between the ALT concentrations and MMSE (*R*^2^ = 0.22, B = 1.99, SE(B) = 0.91, *p* = 0.03), and the AST/ALT ratio and MMSE (*R*^2^ = 0.22, B = −3.01, SE(B) = 1.24, *p* = 0.02) in women. Contrarily, no significant association emerged in men.

Considering women and men according to the presence or absence of dementia, significant associations were found only in women without dementia. In particular, after adjustment for age, FI, and BMI, the multivariate linear regression analyses highlighted significant results between the ALT concentrations and MMSE (*R*^2^ = 0.31, B = 1.84, SE(B) = 0.79, *p* = 0.02), and the AST/ALT ratio and MMSE (*R*^2^ = 0.30, B = −2.18, SE(B) = 1.05, *p* = 0.04).

Categorizing the subjects by diagnosis, age and sex distribution were similar between controls and patients with dementia (Table 2). As expected, the analyses highlighted differences in education, MMSE scores, and FI values between the groups. In particular, the demented patients showed a lower educational level and MMSE score than controls (*p* < 0.001) and were the frailest (*p* < 0.001). Contrarily, no difference was found in the GDS score and BMI (Table 2).

Regarding the liver enzymes, no difference was detected in the AST levels between the groups. To note, the patients with dementia showed lower levels of ALT compared to controls (*p* = 0.005) and, consequently, the highest AST/ALT ratio value (*p* = 0.003) (Table 2).

Considering women in relation to diagnosis, no difference was found in the age, GDS score, and BMI between the groups (Table 3). Women with dementia had a lower educational level and MMSE score compared to controls (*p* = 0.004 and *p* < 0.001, respectively) and were frailer than the other group (*p* < 0.001) (Table 3).

Regarding men in relation to diagnosis, as observed in women, no difference was found in terms of age, GDS score, and BMI (Table 3). Our results showed that demented men were less literate compared to controls (*p* = 0.001) and, as expected, showed MMSE scores significantly lower than control men (*p* < 0.001). Similar to women, men with dementia were frailer than the other group (*p* < 0.001) (Table 3).

Interestingly, regarding the liver enzymes in women, there was no difference in both the AST concentrations and AST/ALT ratio values between the two groups (Table 3), whereas a trend resulted for the ALT serum concentrations (*p* = 0.09).

Differently, in men, the AST did not differ, whereas the ALT and AST/ALT ratio did. Specifically, demented men showed significantly lower levels of ALT (*p* = 0.009) and a higher AST/ALT ratio value (*p* = 0.003) compared to controls (Table 3).

When we compared men and women with the same diagnosis, we showed a lower educational level in control women than men (*p* = 0.001). Women were the most depressed both in controls and demented persons (*p* = 0.03 and *p* = 0.007, respectively). FI was higher in men compared to women in persons with dementia (*p* = 0.02) (Table 3).

Regarding the plasmatic concentrations of transaminases, the ALT were lower, and the AST/ALT ratio was higher in control women than control men (*p* = 0.008 and *p* = 0.001, respectively). No difference was found in the AST and ALT concentrations and the AST/ALT ratio between women and men in persons with dementia (Table 3).

## 4. Discussion

This study analyzed the most diffused serum-based liver function markers in a cohort of older people resident in the community investigated for a suspected cognitive decline, providing several interesting findings.

First, although the serum concentrations of the liver enzymes were within the normal range, a difference was found in the ALT (but not in the AST) levels and the AST/ALT ratio when comparing subjects without cognitive decline and patients with dementia. Specifically, patients suffering from dementia showed significantly lower ALT concentrations and higher AST/ALT ratio values compared to controls. The same differences were found between men with dementia and men without dementia but disappeared in women. Curiously, differences in the hepatic enzyme parameters were discovered between control women and men, whereas no difference resulted between demented women and men.

ALT and AST are the most commonly employed laboratory parameters for screening, diagnosing, or monitoring liver status [50]. The serum concentrations of these enzymes, in particular ALT, are age-dependent [51], and their reduction may be considered a feature of hepatic aging. These enzymes are also correlated with dementia [8,40], as confirmed in our cohort.

Some studies argue that the reduced hepatic synthesis and metabolic function may contribute to or correlate with cerebral hypometabolism, which is recognized as a common feature in demented persons [5,8]. Indeed, a reduction in ALT levels seems to cause a drop in pyruvate production, leading to a reduced gluconeogenesis and, thus, a lower glucose availability as an energy source to various tissues (e.g., brain) [52], inducing a fragilization in older persons [19,21,53]. In a recent study, low levels of plasma aminotransferases and increased enzyme ratios have been associated with brain glucose hypometabolism, particularly in the areas implicated in memory and executive function [8]. Moreover, the variation in ALT and AST concentrations may have an effect on glutamate, an excitatory neurotransmitter of the central nervous system with a role in synaptic transmission [54]. Moreover, a number of studies reported that low serum concentrations of liver enzymes, in particular ALT, are a biomarker for sarcopenia, malnutrition, frailty, and disability [19,20,21,22,23,55].

Interestingly, a recent study reported an age dependence of ALT concentrations in men, whereas this dependence was weaker in women [30]. Moreover, this study also showed that ALT concentrations were reduced in healthy men aged more than 65 years compared to women of the same age [30], not only confirming the strong dependence of liver enzymes concentrations on age but also illustrating sex differences in liver-associated metabolic functions [30]. The lowest ALT and the highest AST/ALT ratio values found in the women in our cohort confirm that sex-specific changes (probably driven by different hormone settings [56]) occur in the liver during aging.

Effectively, the liver can undoubtedly be considered one of the most sexually dimorphic organs, known to have a different essential role in the regulation of energy storage and metabolic fluxes in women and men [57]. However, to date, studies investigating the possible contribution of sex to the relation between liver function and cognitive decline as well as dementia are lacking.

In the women in our study, independently of the presence of dementia, the ALT concentrations and the AST/ALT ratio values showed a positive (ALT) and a negative (ratio) association with the MMSE score. In men, no significant results were obtained, suggesting a role of sex in the occurrence of this association.

In our cohort, women were more numerous than men; therefore, there is the possibility that the lower ALT concentrations and the higher AST/ALT ratio values found in controls than demented patients could be driven by the high percentage of women. Surprisingly, categorizing the subjects by sex, these differences were confirmed only in men and completely disappeared in women. This data could be due to the influence of sex steroids [29,57].

Unexpectedly, when we compared women and men with the same diagnosis, a difference in the hepatic enzyme parameters emerged in cognitively healthy subjects but not in persons with dementia. This is an interesting result, suggesting that sex differences weaken when dementia takes over.

Frailty has been reported to predict incident dementia [58]; therefore, the lowest FI values found in our demented persons were expected. Moreover, the men in our cohort were frailer than the women. This could be due to the high FI values observed in the demented men who were much frailer, not only than control men but also than demented women. Therefore, the high frailty characterizing men with dementia might contribute to raising the FI value of men in the overall cohort.

Our research has some limitations. First, in addition to AST and ALT, we did not consider other liver function markers (e.g., total bilirubin, albumin, alkaline phosphatase) which could help to better describe liver status. Second, AST and ALT are state variables that may be affected by the influence of many other factors not taken into consideration in this study. Third, this is a cross-sectional study that consecutively included community-dwelling older adults from March 2009 to February 2018. This extended period of time could be considered a limitation as well as a strength of the study because it allowed us to collect data from a large number of subjects.

In conclusion, we observed an association between serum aminotransferases and dementia in a cohort of community-dwelling older persons investigated for a suspected cognitive decline. In particular, our findings support attention to sex difference in liver function, suggesting a role in the pathogenesis of dementia.

Based on these considerations, as physical exercise and diet are known lifestyle factors that not only contrast metabolic diseases but also reduce the risk of dementia [59,60], both physical and nutritional interventions could be proposed as powerful means to positively influence liver parameters and reverse their possible negative effect on brain function.

## Figures and Tables

**Table 1:** Characteristics and liver enzyme concentrations in the overall cohort and in subjects categorized by sex.

	Overall Cohort (*n* 419)	Women (*n* 294)	Men (*n* 125)	*p*
Age (years)	79.9 (5.5)	79.8 (5.6)	79.9 (5.0)	0.86
Education (years)	9.1 (4.6)	8.5 (4.4)	10.3 (4.8)	0.001
MMSE score	23.1 (5.6)	23.0 (5.7)	23.3 (5.3)	0.64
GDS score	12.4 (6.7)	13.4 (6.6)	9.6 (6.0)	<0.001
FI	0.28 (0.21–0.37)	0.27 (0.19–0.37)	0.32 (0.23–0.40)	0.02
BMI (kg/m^2^)	25.6 (4.8)	25.4 (5.1)	26.2 (3.9)	0.09
AST (U/L)	19.0 (16.0–22.0)	19.0 (16.0–22.0)	19.0 (16.0–23.5)	0.28
ALT (U/L)	15.0 (11.0–19.0)	14.0 (11.0–18.0)	15.0 (12.0–20.0)	0.007
AST/ALT ratio	1.31 (1.08–1.54)	1.33 (1.15–1.55)	1.23 (1.04–1.46)	0.003

Education was indicated as years of schooling; MMSE: mini-mental state examination; GDS: geriatric depression scale; FI: frailty index; BMI: body mass index; AST: aspartate aminotransferase; ALT: alanine aminotransferase.

**Table 2:** Characteristics and liver enzyme concentrations in subjects categorized by diagnosis.

	Controls (*n* 171)	Dementia (*n* 248)	*p*
Age (years)	79.5 (5.7)	80.1 (5.3)	0.22
Sex (% women)	69.6%	70.6%	0.46
Education (years)	10.2 (4.5)	8.2 (4.4)	<0.001
MMSE score	27.0 (3.4)	20.4 (5.2)	<0.001
GDS score	12.4 (6.6)	12.5 (6.8)	0.97
FI	0.24 (0.17–0.32)	0.32 (0.24–0.41)	<0.001
BMI (kg/m^2^)	26.1 (5.0)	25.3 (4.6)	0.13
AST (U/L)	19.0 (16.0–23.0)	19.0 (16.0–22.0)	0.42
ALT (U/L)	15.0 (12.0–20.0)	14.0 (11.0–18.0)	0.005
AST/ALT ratio	1.26 (1.05–1.47)	1.36 (1.12–1.60)	0.003

Education was indicated as years of schooling; MMSE: mini-mental state examination; GDS: geriatric depression scale; FI: frailty index; BMI: body mass index; AST: aspartate aminotransferase; ALT: alanine aminotransferase.

**Table 3:** Characteristics and liver enzyme concentrations in controls and patients with dementia categorized by sex.

	Women			Men		
	Controls (*n* 119)	Dementia (*n* 175)	*p*	Controls (*n* 52)	Dementia (*n* 73)	*p*
Age (years)	79.5 (5.9)	80.1 (5.4)	0.37	79.4 (5.3)	80.3 (4.8)	0.35
Education (years)	9.4 (4.2) ^a^	7.9 (4.4)	0.004	12.0 (4.8)	9.0 (4.4)	0.001
MMSE score	27.1 (3.3)	20.2 (5.3)	<0.001	26.8 (3.6)	21.0 (4.9)	<0.001
GDS score	13.3 (6.6) ^b^	13.6 (6.8) ^c^	0.79	9.9 (6.1)	9.2 (6.0)	0.70
FI	0.23 (0.17–0.31)	0.30 (0.23–0.40) ^d^	<0.001	0.26 (0.19–0.34)	0.35 (0.26–0.43)	<0.001
BMI (kg/m^2^)	26.1 (5.6)	24.9 (4.7)	0.07	26.2 (3.0)	26.3 (4.4)	0.88
AST (U/L)	19.0 (16.0–22.0)	18.0 (16.0–22.0)	0.42	19.0 (16.0–24.0)	19.0 (16.0–22.0)	0.77
ALT (U/L)	15.0 (12.0–18.0) ^a^	13.0 (10.0–18.0)	0.09	17.0 (14.0–23.0)	14.0 (12.0–19.0)	0.009
AST/ALT ratio	1.31 (1.11–1.50) ^a^	1.36 (1.15–1.60)	0.11	1.14 (0.97–1.32)	1.31 (1.06–1.60)	0.003

Education was indicated as years of schooling; MMSE: mini-mental state examination; GDS: geriatric depression scale; FI: frailty index; BMI: body mass index; AST: aspartate aminotransferase; ALT: alanine aminotransferase. ^a^ *p* < 0.01 vs. control men, ^b^ *p* = 0.03 vs. control men, ^c^ *p* = 0.007 vs. men with dementia, ^d^ *p* = 0.02 vs. men with dementia

## Data Availability

The data presented in this study are available on request from the corresponding author.

## Supplementary Materials

The following supporting information can be downloaded at: https://www.mdpi.com/article/10.3390/nu14234973/s1, Table S1: List of the 47 biochemical and health deficits included in the FI.
