# Supplementary material for: Liver Enzymes in a Cohort of Community-Dwelling Older Persons: Focus on Sex Contribution
Source: Nutrients. 2022 Nov 23;14(23):4973. doi: 10.3390/nu14234973 (PMC9737251; doi:10.3390/nu14234973)
Supplement: Supplementary file 1 [file nutrients-14-04973-s001.zip › nutrients-1999760-supplementary.pdf]

Table S1. List of the 47 biochemical and health deficits included in the FI.

Deficit
**Biochemical parameters**
Cholesterol ≤200 mg/dL
CRP <0.5 mg/dL
Vitamin B12 191-663 ng/L
Folate 4.6-18.7 μg/L
25-OH Vitamin D >30 μg/L
TSH 0.28-4.30 mIU/L
**Signs**
Pain
Bowel incontinence
Sleep disorders
BMI 21-30 kg/m^2
Edema
Tremor
**Disabilities**
Mobility impairment
ADL - disability in self-feeding
ADL - disability in dressing
ADL - disability in bathing
ADL - disability in transferring
ADL - disability in toileting
ADL - incontinence
IADL - disability in using telephone
IADL - disability in shopping
IADL - disability in food preparation
IADL - disability in housekeeping
IADL - disability in doing laundry
IADL - disability in travelling by car or public transportation
IADL - disability in medication use
IADL - disability in handling finances
**Diseases**
Hypertension
Diabetes
Congestive heart failure
Coronary heart disease
Cardiac arrhythmia
Chronic obstructive pulmonary disease
Decreased visual acuity
Hearing loss
Osteoarthritis
Vascular endothelial abnormalities
Chronic renal insufficiency
Hepatopathy
Depression
Cerebrovascular disease
Cognitive impairment
Cancer

Osteoporosis
Anemia
Diverticulosis
Mild cognitive impairment

CRP: C-Reactive Protein, TSH: Thyroid-Stimulating Hormone, BMI: Body Mass Index, ADL: Activity of Daily Living, IADL: Instrumental Activity of Daily Living.
